# Uric acid enhances the antitumor immunity of dendritic cell-based vaccine

**DOI:** 10.1038/srep16427

**Published:** 2015-11-10

**Authors:** Yihan Wang, Xuelei Ma, Chao Su, Bin Peng, Jing Du, Hongyuan Jia, Min Luo, Chunju Fang, Yuquan Wei

**Affiliations:** 1 State Key Laboratory of Biotherapy and Cancer Center, West China Hospital, Sichuan University, and Collaborative Innovation Center for Biotherapy, Chengdu, China

## Abstract

Uric acid (UA) released from dying cells has been recognized by the immune system as a danger signal. In response to UA, dendritic cells (DC) in the immune system mature and enhance the T cell response to foreign antigens. It is conceivable that the antitumor immunity of a tumor vaccine could be promoted by the administration of UA. To test this concept, we applied UA as an adjuvant to a DC-based vaccine, and discovered that the administration of UA as an adjuvant significantly enhanced the ability of the tumor lysate-pulsed DC vaccine in delaying the tumor growth. The antitumor activity was achieved with adoptively transferred lymphocytes, and both CD8^+^ T cells and NK cells were required to achieve effective immunity. This resulted in an increased accumulation of activated CD8^+^ T cells and an elevated production of IFN-γ. Collectively, our study shows that the administration of UA enhances the antitumor activity of tumor lysate-pulsed DC vaccine, thus providing the preclinical rationale for the application of UA in DC-based vaccine strategies.

Malignant tumors have become a major problem in public health. Although conventional chemotherapy remains the backbone of current treatment, it is still hindered by various side effects and developmental limitations^1^,^2^,^3^. Recent scientific advances have enhanced our understanding of the immune system and its role in attacking malignant cells. The tumor vaccine has evolved as an alternative treatment modality as it harnesses the body’s natural ability to recognize and eliminate cancer cells from the body. An effective tumor vaccine must possess the ability to induce a safe, antigen-specific and long-lasting immune response. In this regard, the use of dendritic cells (DC) in vaccine strategies has attracted a fair amount of attention.

DC, derived from hematopoietic progenitor cells, are known as the “sentinels” of the immune system. They are the most powerful antigen presenting cells with the ability to elicit both primary and secondary immune responses to foreign antigens^4^,^5^. Immature DC firstly internalize and process antigens in peripheral tissues and then migrate to draining lymphoid organs, where they undergo maturation. Subsequently, they stimulate T cells through the up-regulation of cell-surface major histocompatibility complexes (MHC) and costimulatory molecules^6^,^7^. Immunization via DC loaded with tumor-associated antigens could potentially be a powerful strategy of inducing specific antitumor immunity. The usage of tumor lysates as a possible source of tumor-associated antigens is found to have several benefits, including mimicking the physiologic processes of tumor recognition and rejection^8^. In present studies, several groups pulsed tumor lysates onto bone marrow-derived DC and used them for immunization in experimental animals. They have indicated that the tumor lysates-pulsed DC (TP-DC) vaccine is a powerful strategy of eliciting broader T cell immune responses^8^,^9^,^10^. However, this DC-based vaccine, although offering considerable advantages, still has a limited antitumor effect in some cases, thus requiring the addition of adjuvants to elicit a strong and long-lasting immune response.

The “danger model” has proposed that antigen-presenting cells are activated by danger or alarm signals from injured cells, thus promoting T cell responses to copresented antigens^11^. Recently, uric acid (UA) released from dying cells has been identified as a danger signal for the immune system^12^,^13^,^14^,^15^. UA is a natural product of the purine metabolic pathway and it is released from dying cells, which eventually leads to crystallization. It was reported that crystalline UA stimulates the maturation of DC by increasing the expression of costimulatory molecules CD80 and CD86, and enhances T cell responses to foreign antigens^14^,^16^. Crystalline UA has been shown to activate macrophages to produce inflammatory mediators^11^, and it may stimulate DC in a similar way. It is conceivable that UA, an endogenous danger signal, could be used as a potential adjuvant to DC-based vaccines for potent antitumor immunity.

Since we have been exploring approaches to enhance the protective and therapeutic activities of DC-based vaccines, we combined the administration of TP-DC and UA based on these existing studies. Collectively, our results demonstrated that UA enhanced the antitumor activity of TP-DC vaccine against tumors.

## Results

### Induction of protective and therapeutic antitumor immunity

**Figure 1:**
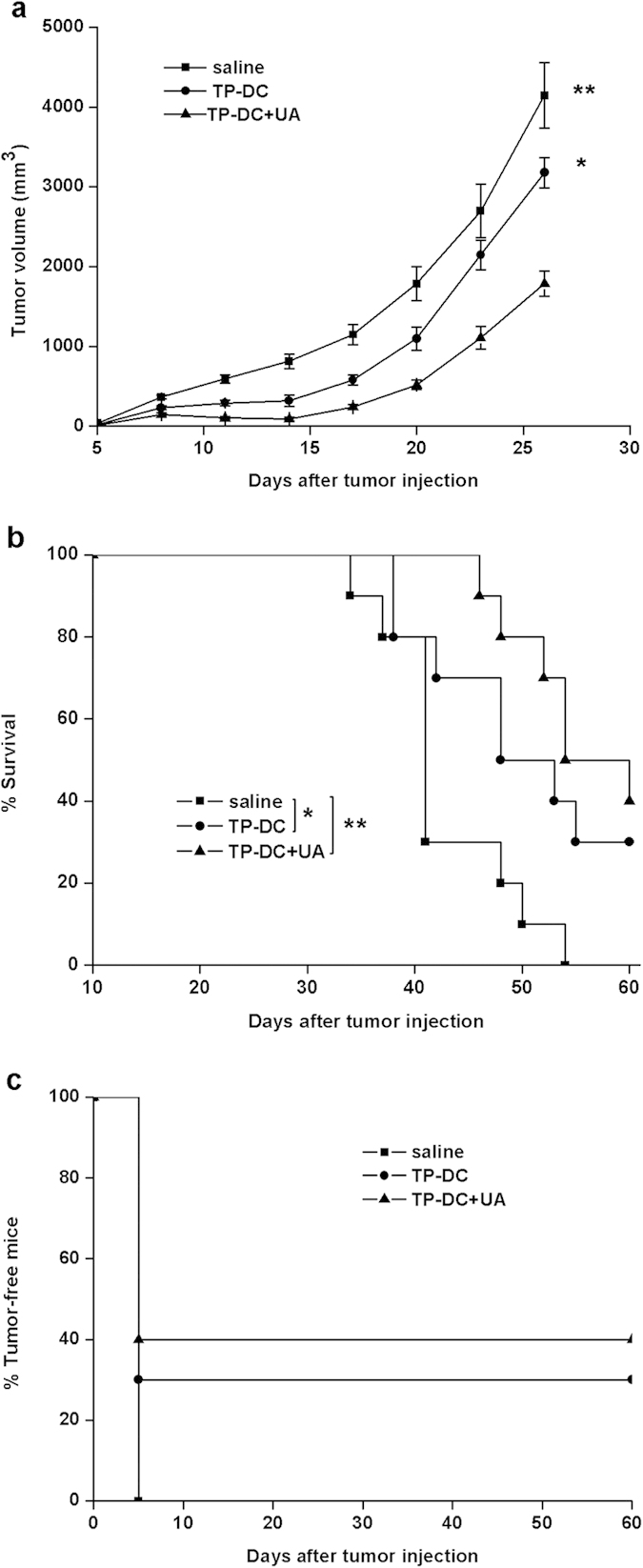
Induction of protective antitumor immunity. Mice (10 mice in each group) were immunized subcutaneously with tumor lysate-pulsed DC together with 100 μg UA (TP-DC+UA), tumor lysate-pulsed DC (TP-DC) and saline on day 0, 14, 21. Mice were then challenged with 3 × 10^6^ E.G7 cells subcutaneously 1 week after the third immunization. a, Tumor volumes (mean ± SEM) of mice in each group were shown. b, Percentage survival of mice treated. The survival rate was 40% and 30% for mice immunized with TP-DC+UA and TP-DC respectively at day 150. c, Percentage tumor-free mice in each group. (*p < 0.05; **p < 0.01).

To investigate protective antitumor immunity, we immunized mice with TP-DC together with 100 μg UA, TP-DC or saline on day 0, 14, 21 and challenged them with tumor cells at day 7 after the third immunization. Tumor growth inhibition was determined by the size of tumor. As shown in Fig.1a, in E.G7 T lymphoma model, immunization using TP-DC with 100 μg UA significantly inhibited the tumor growth compared to using TP-DC alone and the saline control groups. In addition, the percentage of tumor-free mice using TP-DC with 100 μg UA and TP-DC alone was 40% and 30%, respectively, but the group immunized with saline had 100% tumor-bearing mice (Fig. 1b). Although the survival time was not significantly different between TP-DC and TP-DC with 100 μg UA, they were both significantly longer than that of the saline group (Fig. 1c). The dose of UA used in the experiment is based on previous studies^11^,^14^. We applied 20 μg, 100 μg and 200 μg UA as an adjuvant to TP-DC vaccine respectively. We found that UA was ineffective at the 20 μg dose. In addition, 100 μg and 200 μg led to similar tumor suppression and overall survival. Therefore, we chose 100 μg as an optimal dose in our experiment.

**Figure 2:**
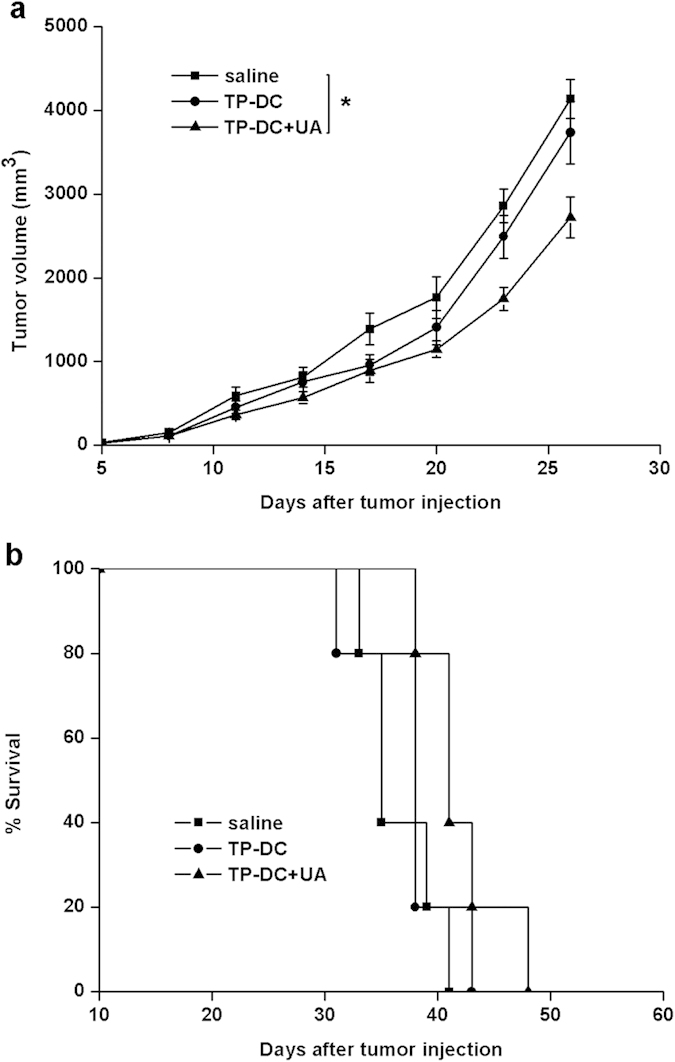
Induction of therapeutic antitumor immunity. Mice (5 mice in each group) were treated subcutaneously with TP-DC+UA, TP-DC and saline twice weekly for seven times starting at day 4 after 3 × 10^6^ E.G7 cells were introduced subcutaneously into mice. a, Tumor volumes (mean ± SEM) of mice in each group were shown. b, Percentage survival of mice treated. (*p < 0.05).

The therapeutic efficacy of the vaccine was next explored in the established tumor model. We treated mice on day 4 after the injection of E.G7 cells and twice weekly for seven times. Treatment with TP-DC together with 100 μg UA showed smaller tumor volume compared with that of the controls (Fig.2a). The difference in tumor volume was only significant between TP-DC with 100 μg UA and the saline group. However, the survival time was not significantly different between groups (Fig. 2b).

We have investigated the potential long-term toxicity of the immunized mice. No adverse changes were found in gross measures such as ruffling of fur, weight loss or life span.

### Adoptive transfer of T cells or immunoglobulins and their antitumor efficacy

**Figure 3:**
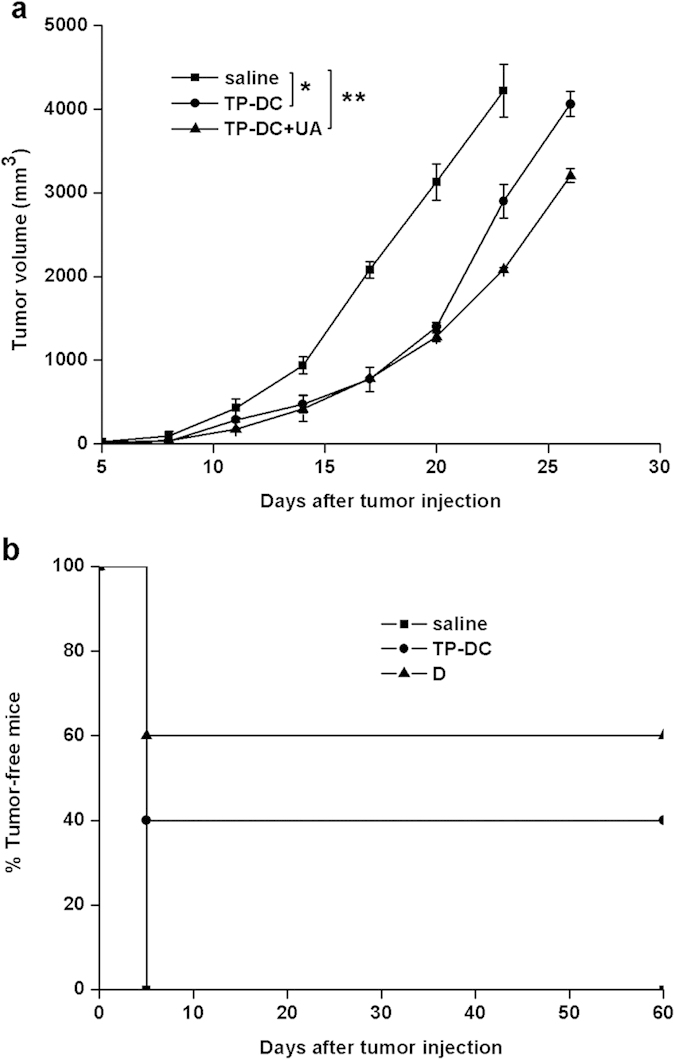
Adoptive transfer of T cells *in vivo*. **(a**) T cells were isolated from mice immunized with TP-DC+UA, TP-DC and saline, respectively. The adoptive transfer of 1 × 10^7^ T cells from mice immunized with TP-DC+UA showed more effective antitumor activity than controls (n = 5). (**b**) Percentage tumor-free mice in each group. (*p < 0.05; **p < 0.01).

In order to explore the underlying mechanisms by which the antitumor activity was induced by TP-DC with 100 μg UA, we adoptively transferred T cells or immunoglobulins isolated from TP-DC with 100 μg UA-immunized mice and control mice to recipient mice. The treatment with T cells from TP-DC alone or with 100 μg UA provided effective protection from tumor growth compared with the saline group (Fig. 3a). By contrast, the tumor-free rate of mice immunized with TP-DC with 100 μg UA and TP-DC alone was 60% and 40%, respectively, although there was no statistical significance between tumor volume of the two groups (Fig. 3b). In addition, adoptive transfer of immunoglobulins from mice immunized with TP-DC with 100 μg UA did not result in protection from tumor growth compared with the controls. The result indicated that cell immunization might play a critical role in the antitumor activity induced by TP-DC with 100 μg UA.

**Figure 4:**
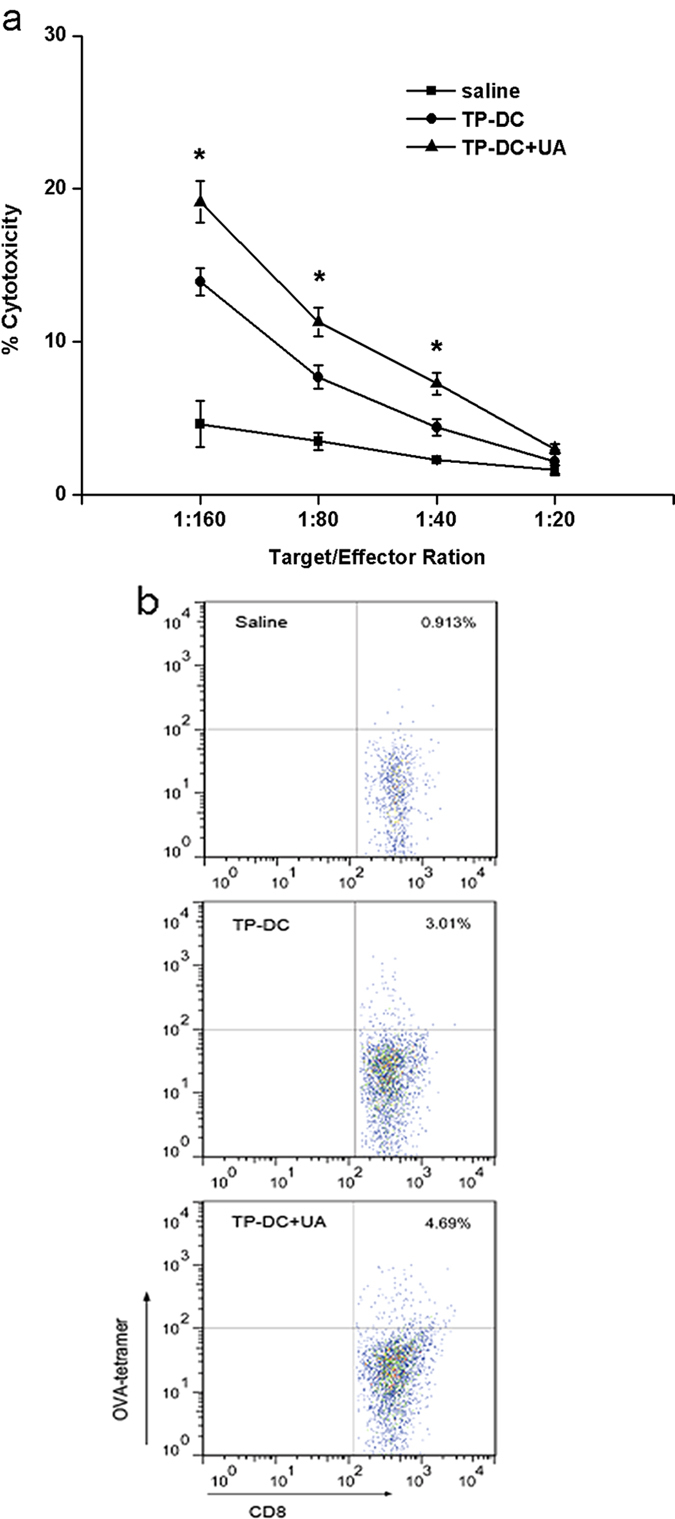
Experiment of CTL cytotoxicity mediated by tumor-specific T cells. **(a**) T cells derived from spleens of TP-DC+UA, TP-DC and saline immunized mice were tested against E.G7 cells at different E:T ratios by a standard 4 h^51^Cr release assay as described in Materials and Methods. T cells from the spleens of TP-DC+UA immunized mice showed higher cytotoxicity against E.G7 cells than did T cells from TP-DC or saline (n = 3). Data were shown as mean ± SEM. (**b**) The generation of tetramer^+^ CTLs was determined by flow cytometry using FITC-conjugated anti-CD8 mAb and PE-conjugated H-2k^b^–OVA_257–264_ peptide tetramer. (*p < 0.05; **p < 0.01).

Next, the tumor killing activity of CTLs was explored. T cells isolated from mice immunized with TP-DC with 100 μg UA showed significantly stronger cytotoxicity against E.G7 tumor cells than those from the control groups (Fig. 4a).

Then we wanted to investigate whether our vaccine induced the development of OVA-specific CTLs. As shown in Fig. 4b, vaccination of mice using TP-DC with 100 μg UA induced increased frequency of OVA-tetramer^+^ CD8^+^ T cells. The finding indicated that the vaccine induced tetramer^+^ CD8^+^ CTLs that specifically targeted E.G7 tumor cells by recognition of H-2k^b^-binding OVA_257–264_ peptide antigen.

### Role of CD8^+^ T cells and NK cells in the induction of antitumor activity

**Figure 5:**
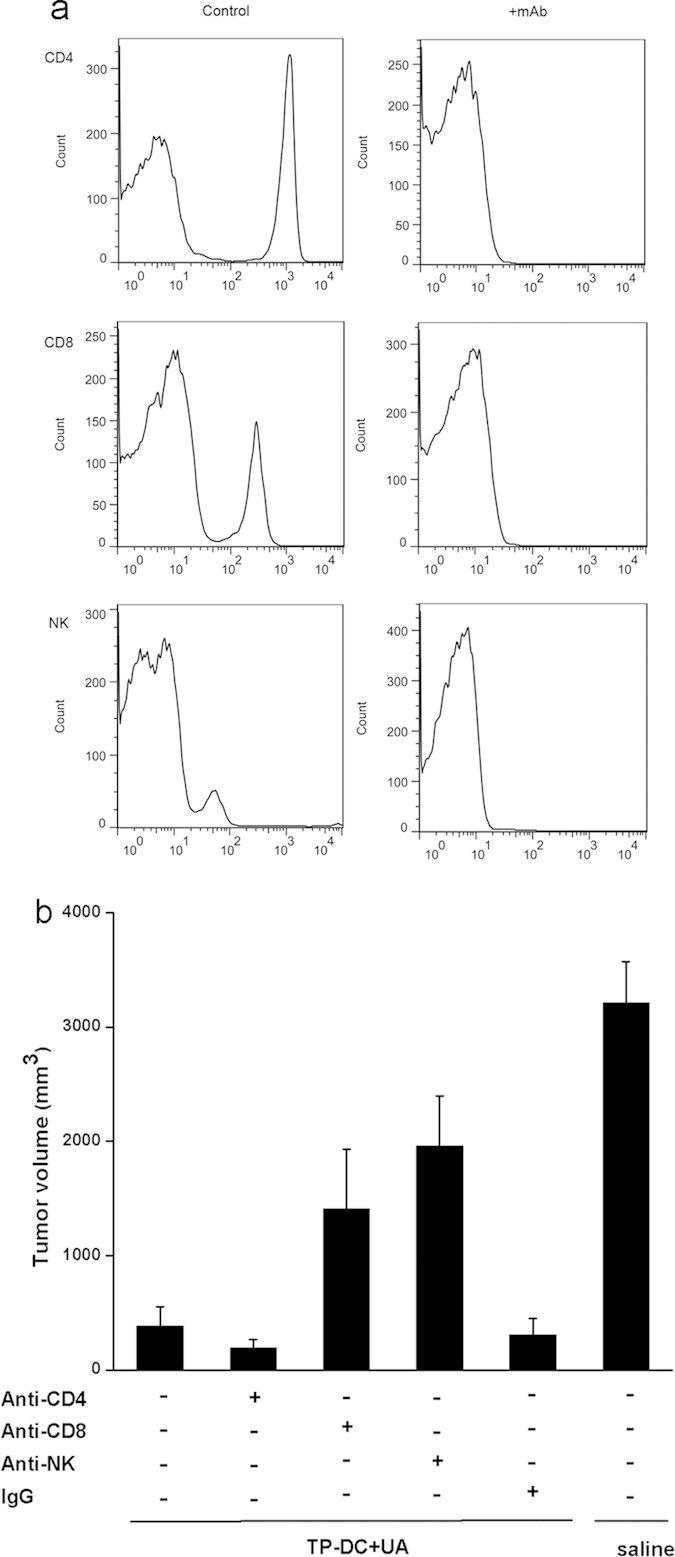
Abrogation of antitumor activity by *in vivo* depletion of immune cell subsets. Mice were immunized and then challenged with E.G7 cells. Depletion of immune cell subsets was described in Materials and Methods. (**a**) The efficacy of antibody depletion was detected by flow cytometry. (**b**) Depletion of CD8^+^ T cells and NK cells both showed partial abrogation of the antitumor activity with the immunization of TP-DC+UA. The results represent at day 17 after tumor injection. Similar results can be found at other time points. Data were shown as mean ± SEM. (*p < 0.05; **p < 0.01).

To further explore the roles of immune cell subsets in antitumor activity induced by TP-DC with 100 μg UA, we depleted CD4^+^ T cells, CD8^+^ T cells and NK cells by injecting the corresponding monoclonal antibodies. The depletion of CD4^+^, CD8^+^ and NK cells were determined by flow cytometry (Fig. 5a). The results indicated that *in vivo* depletion of CD8^+^ T cells and NK cells could both partially abrogate the antitumor activity using the immunization of TP-DC with 100 μg UA, whereas the depletion of CD4^+^ T cells failed to abolish the antitumor activity (Fig. 5b). In addition, the treatment with normal rat IgG showed no effect. These data suggest that both CD8^+^ T cells and NK cells may play important roles in the antitumor activity using the vaccination with TP-DC with 100 μg UA.

### Cytokine production by spleen cells

**Figure 6:**
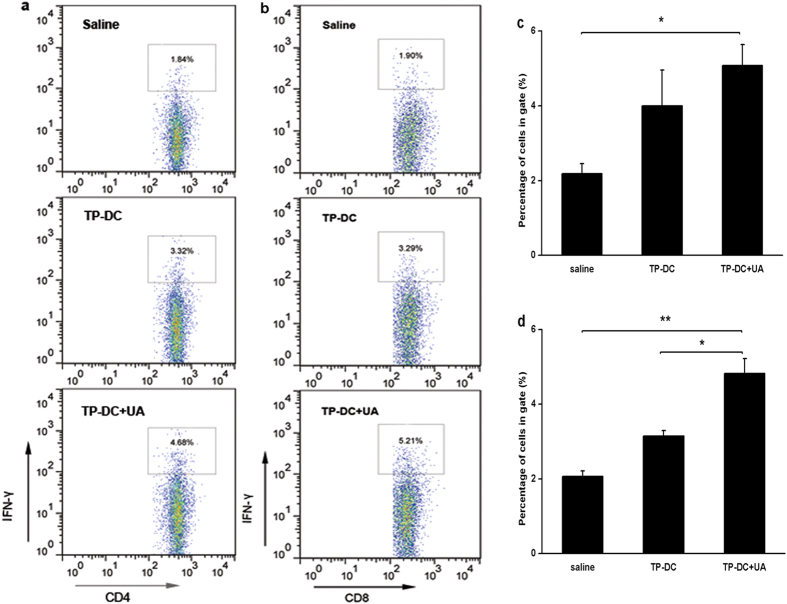
Activation of CD8^+^ T cells *in vitro*. T cells isolated from spleens of mice immunized with TP-DC+UA, TP-DC and saline were stimulated with OVA_257–264_ or OVA_323–339_ for 24 h and added with Golgi Stop during the last 4–6 h. Percentage of the IFN-γ expressing CD4^+^ T cells (**a,c**) and IFN-γ expressing CD8^+^ T cells (**b,d**) were analyzed by flow cytometry (n = 3). Data were shown as mean ± SEM. (*p < 0.05; **p < 0.01).

**Figure 7:**
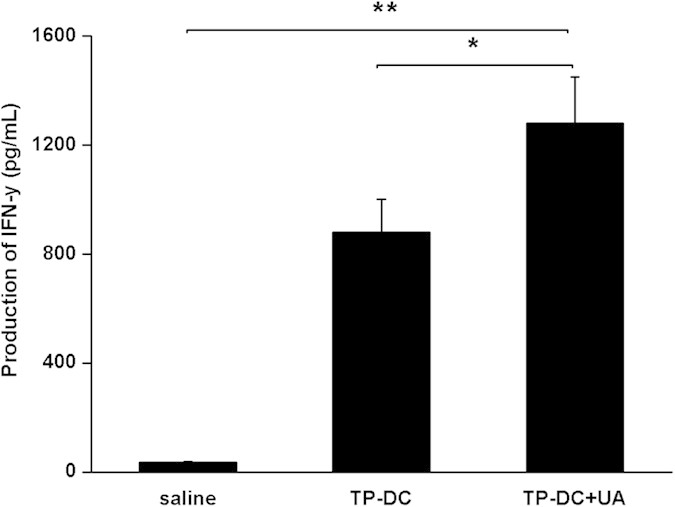
Cytokine production by spleen cells. Spleen cells isolated from mice immunized with TP-DC+UA, TP-DC and saline were stimulated with OVA_257–264_ or OVA_323–339_ for 48 h *in vitro*. Supernatants in each group were harvested and assayed for IFN-γ production (n = 3). Data were shown as mean ± SEM. (*p < 0.05; **p < 0.01).

We performed the *in vitro* study to verify the specific activation of T cells. The OVA_257–264_ or OVA_323–339_ stimulated T cells were stained by anti-CD4-APC and anti-IFN-γ-PE or anti-CD8-FITC and anti-IFN-γ-PE. Flow cytometric analysis showed that the number of activated CD8^+^ T cells (CD8^+^, IFN-γ^+^) in mice immunized with TP-DC with 100 μg UA had a significant increase compared with the controls, whereas the activated CD4^+^ T cells (CD4^+^, IFN-γ^+^) were observed with less increase in the immunized group (Fig. 6a,b). Statistical analysis also revealed the same tendency as described in Fig. 6c,d. In addition, the cell-free culture supernatants of spleen cells were harvested and assayed for IFN-γ activity in response to OVA_257–264_ re-stimulation. The production of IFN-γ in mice immunized with TP-DC with 100 μg UA was significantly greater than that in mice immunized with TP-DC, while spleen cells from mice immunized with saline alone produced a few IFN-γ (Fig. 7).

## Discussion

DC are professional antigen presenting cells playing a vital role in the initiation, programming and regulation of tumor-specific immune responses^17^,^18^. Immature DC capture and process antigens in peripheral tissues, and mature DC migrate to lymphoid organs where they prime T cell immune responses^7^,^19^,^20^. Due to these properties, efforts have been made on the utilization of the immunostimulatory power of activated DC. In fact, a number of studies have shown that immunization by DC loaded with tumor antigens can prime a tumor specific CTL response and induce protective antitumor immunity in experimental animals^8^,^9^,^10^,^21^,^22^,^23^,^24^. Furthermore, DC immunotherapy has already been introduced in the clinic, which has been proved feasible, safe and effective in some patients^25^,^26^,^27^,^28^,^29^. However, further investigations are still required to improve the immune response of DC-based vaccines.

UA, an end product of purine catabolism, has been documented recently to act as a danger signal for the immune system^12^,^13^,^14^,^15^. Shi and colleagues have shown that UA stimulates DC maturation *in vitro* by increasing the expression of the costimulatory molecules CD80 and CD86^14^. Additionally, it enhances the generation of CD8^+^ T cell responses when co-injected with a particulate antigen. The administration of allopurinol or uricase, which markedly decreased plasma UA concentrations, was shown to substantially inhibit this T cell priming. The concentrations of UA that stimulated these DC corresponded to the one at which UA crystals were precipitated. It was shown that preformed crystals were highly stimulatory, whereas soluble UA was not^11^,^14^,^16^. Other groups are now corroborating the stimulatory effects of UA. Hu *et al.* demonstrated that UA levels were elevated in tumors undergoing immune rejection^11^. Behrens *et al.* indicated that UA could enhance antibody immunity^30^. Recently, Kuhn *et al.* combined UA and *Mycobacterium Smegmatis* for local treatment, which was effective at delaying tumor growth^31^. These findings indicate the adjuvant effects of UA and encourage the potential administration of UA in tumor vaccines.

Several observations have been made in the present study concerning the antitumor immunity of tumor-lysates based DC vaccines^8^,^9^,^10^. In addition, crystalline UA has been shown to activate innate immune effectors including macrophages and DC^32^. The present study has, to our knowledge, first demonstrated that the administration of UA as an adjuvant could augment the *in vivo* antitumor effects of the TP-DC vaccine. T cell effectors, other than autoantibodies, were involved in the antitumor activity. In addition, our study suggests autoreactive immune response against the tumor cells may be provoked in cross-reaction by the immunization of TP-DC with UA. In this process, exogenous antigens cross over to endogenous pathway to gain access to MHC class I. In our study, the successful generation of MHC class I-dependent CD8^+^ CTL activity was observed in cytotoxicity assays *in vitro*. We further showed enhanced accumulation of tumor-specific OVA-tetramer^+^ CD8^+^ CTLs with our vaccine. In addition, this antitumor activity could be achieved with adoptively transferred lymphocytes, and the depletion of both CD8^+^ T cells and NK cells partially eliminated the anti-tumor activity *in vivo*. These findings suggest that MHC class I-dependent CD8^+^ CTL-mediated immune responses may play an important role in the antitumor activity of the TP-DC with UA vaccine, and that NK cells are also involved at the effector phase.

In this study, successful immunotherapy resulted in the activation of both CD8^+^ T cells and NK cells. Accordingly, the vaccine may have broad antitumor effects against tumors that are sensitive to the CD8^+^ T cell or NK cell-mediated killing. However, CD4^+^ T cells elicited little, if any, function in this vaccine. During the *in vitro* experiment, lymphocytes stimulated with OVA_257–264_ showed significantly increased number of CD8^+^ T cells producing IFN-γ. Furthermore, ELISA also observed increased production of IFN-γ in the lymphocyte culture supernant. These results further supported the important role of CD8^+^ T cells in this vaccine. In addition, there might be an efficient cross talk between NK cells and DC. DC are documented to strongly augment the cytotoxic function of NK cells^33^,^34^. In turn, NK cells upregulate the expression of costimulatory molecules on DC and the production of IL-12 and IL-15, thus shaping adaptive immunity toward a Th-1 response^35^. A similar cooperation between CD8^+^ T cells and NK cells was also observed in models of local immunotherapy with immune-activating agents^31^,^36^,^37^. In our study, purified immunoglobulins did not show any antitumor effect, and thus CD8^+^ T cell and NK cell-mediated killing were the key processes involved in antitumor activity. However, the underlying mechanism by which UA could enhance the antitumor effect of DC-based vaccine still needs to be investigated.

Recently, several clinical trials have indicated the ability of TP-DC to induce antitumor T cell responses in patients with lymphoma^38^,^39^, malignant melanoma^40^, ovarian cancer^41^ and renal cell carcinoma^42^. Therefore, immunoadjuvants are designed to facilitate the mobilization and maturation of DC for enhanced antitumor immunity. UA is an endogenous molecule produced in the end of purine catabolism, which is safe as an adjuvant to the DC vaccine. It is well known that the precipitation of UA in joints can cause gouty arthritis. Therefore, the dose of UA administrated in each vaccine is of crucial importance. The dose we used in this study was safe according to the previous studies^11^,^14^, and no autoimmunity was observed. The results in our study provide preclinical rationale for the use of UA in DC-based vaccine for tumor treatment.

In conclusion, we have demonstrated that UA might serve as an effective adjuvant for DC-based vaccine against tumors. Our findings provide a novel strategy of tumor vaccine for cancer therapy.

## Materials and Methods

### Mice and Tumor model

Wild-type female C57BL/6 mice, aged 6–8 weeks, were purchased from the Beijing HFK Bioscience Co. Ltd. (HFK) and held under specified pathogen free conditions. E.G7 (OVA-transfectant of EL4 murine T lymphoma cells) T lymphoma was established in C57BL/6 mice.

### Preparation of Monosodium Urate Crystal

UA (Sigma) was dissolved at a concentration of 5 mg/mL in 0.1 mol/L sodium borate buffer (pH 8.5). The solution was warmed to 55 °C, and the supernatant after filtering was left to sit for more than 72 h. When monosodium urate crystals formed, they were washed with alcohol and acetone^11^,^14^.

### Generation of TP-DC

TP-DC were prepared as described previously^10^,^43^. Bone-marrow cells were collected from the femur and tibiae of mice and were cultured in complete RPMI1640 (RPMI 1640 with 10% inactivated fetal calf serum (FBS), 2 mmol/L L-glutamine, 100 U/mL penicillin G and 100 μg/mL streptomycin) supplemented with 10 ng/mL GM-CSF (PeproTech, Rocky Hill, NJ) and 10 ng/mL IL-4 (PeproTech, Rocky Hill, NJ) at a starting concentration of 2 × 10^6 ^cells per mL. On day 3, 5, 7, half of the medium was replaced with a fresh medium. Tumor cells were collected and suspended at a concentration of 1 × 10^7^ per mL in complete RPMI1640, followed by >4 cycles of rapid freezing (−180 °C) and thawing (37 °C). The tumor cell suspension was then centrifuged at low speed (400 rpm for 10 min). The supernatant (tumor lysates) was harvested and incubated with purified DC overnight on day 7 at a ratio of 3:1 tumor cells to DC, and then cells were incubated with 1 μg/mL lipopoliaccharide (Sigma). On day 9, TP-DC were collected for further studies.

### Vaccination protocol

Totally 1 × 10^6^ TP-DC resuspended in 100 μL saline together with 100 μg UA were injected subcutaneously on day 0, 14, 21. The mice immunized with TP-DC (1 × 10^6^) alone or saline were used as controls. E.G7 cells were cultured in RPMI medium 1640 with 10% FBS and 0.4 mg/mL G418 (MP Biomedicals). 3 × 10^6^ E.G7 cells were injected subcutaneously into the flank on day 7 after the third immunization. Tumor dimensions were measured every three days with calipers, and tumor volumes were calculated as the product of width^2^ × length × 0.52.

### Adoptive Transfer of T Cells or Immunoglobulins *In Vivo*

Preparation of spleen lymphocytes was performed as previously reported^44^. Briefly, the immunized or control mice were killed on day 7 after the third immunization. Single-cell suspensions were obtained from spleens through a 70-μm nylon mesh filter (BD Biosciences), and lymphocytes were enriched by lymphocyte separation medium (Dakewe Biotech Company) according to the manufacturer’s instructions. To assess the efficacy of T cells in antitumor *in vivo*, spleen lymphocytes (1 × 10^7^) from donor mice on day 7 after the third immunization were adoptively transferred intravenously into recipient mice one day before and three days after the tumor inoculation (3 × 10^6^). Immunoglobulins were purified from the pooled sera derived from the immunized or control mice by affinity chromatography (CM Affi-gel Blue Gel Kit; Bio-Rad). To assess the efficacy of Immunoglobulins in antitumor activity *in vivo*, the purified Immunoglobulins (50 mg/kg) were adoptively transferred intravenously one day before mice were challenged with 3 × 10^6^ tumor cells and then treated twice per week for 3 weeks.

### Depletion of Immune Cell Subsets *In Vivo*

Immune cell subsets were depleted as described previously^45^,^46^. Mice received intraperitoneal injections of 500 μg of either the anti-CD4 (clone GK1.5, rat IgG), anti-CD8 (clone 2.43, rat IgG), anti-NK (clone PK136) mAb or isotype controls one day before the first immunization, and then twice per week for three weeks. Mice were challenged with tumor cells (E.G7) after the third immunization. These hybridomas were obtained from American Type Culture Collection.

### Assay of CTL Cytotoxic Activity *in vitro*

A 4-h^51^Cr release assay was performed as previously described by other reports^8^,^47^. Briefly, spleen lymphocytes obtained from the immunized or control mice, as the effector cell, were treated with ammonium chloride-potassium lysing buffer to deplete erythrocytes. E.G7 (about 1 × 10^6^), as the target cells, were labeled with 100 μCi ^51^Cr for 1 h at 37 °C, and then washed and resuspended at a concentration of 1 × 10^5^ cells/mL. A total of 200 μL of effector cells and ^51^Cr-labeled target cells were assigned at different E:T ratios to each well of 96-well plate and incubated for 4 h at 37 °C. Hundred microliters of supernatant were then harvested, and the activity was calculated by the formula: % cytotoxicity = [(experimental release − spontaneous release)/(maximum release − spontaneous release)] × 100.

### Flow Cytometry

Spleen cells from the mice about 7 days after the third immunization were separated by lymphocyte separation medium and resuspended. OVA-specific CTLs were generated by stimulating spleen lymphocytes with 50 μg/mL of OVA protein for 3 days. The cells were then stained by FITC-conjugated anti-CD8 (BD Biosciences) and PE-conjugated H-2K^b^/OVA_257–264_ complex (MBL Co., Ltd., Nagoya, Japan). For intracellular cytokine staining, spleen T cells were incubated at a density of 2 × 10^6^ cells per mL in complete RPMI 1640 containing 10 μg/mL OVA_257–264_ or OVA_323–339_ for 24 h, and added with Golgi Stop (BD Biosciences) during the last 4–6 h. The cells were then harvested and stained by anti-CD4-APC and anti-IFN-γ-PE or anti-CD8-FITC and anti-IFN-γ-PE (BD Biosciences). Fluorescence profiles were acquired on a FACScan flow cytometer (Becton Dickinson) and analyzed using CellQuest software.

### Cytokine response

Spleen cells from immunized mice 7 days after the third immunization were depleted of erythrocytes. The cells were then resuspended with complete RPMI 1640 and seeded into 24-well microtitration plates at a density of 2 × 10^6^ per well in 1 mL culture medium supplemented with 10 μg/mL OVA_257–264_ or OVA_323–339_ (*in vivo* Gen). After 48 h at 37 °C, the cell-free supernatant was harvested and tested for IFN-γ by ELISA kit (eBioscience Inc.) according to the manufacturer’s instructions. The sensitivity limits for the assays are 0.7 pg/mL.

### Statistical Analysis

All data were presented with mean ± SEM. Statistical analyses were performed using SPSS 19.0 software. Statistical significance was assessed by ANOVA with the Bonferroni post hoc test. Survival data were analyzed using log-rank test. p < 0.05 was regarded as statistically significant.

### Ethics statement

All studies involving mice were approved by the institute’s animal care and use committee. All animal feeding and experiments were carried out in accordance with the guidelines of the institute’s animal care and use committee.

## Additional Information

**How to cite this article**: Wang, Y. *et al.* Uric acid enhances the antitumor immunity of dendritic cell-based vaccine. *Sci. Rep.* **5**, 16427; doi: 10.1038/srep16427 (2015).
